# Use of virus‐induced gene silencing to characterize genes involved in modulating hypersensitive cell death in maize

**DOI:** 10.1111/mpp.12999

**Published:** 2020-10-10

**Authors:** Colin Murphree, Saet‐Byul Kim, Shailesh Karre, Rozalynne Samira, Peter Balint‐Kurti

**Affiliations:** ^1^ Department of Entomology and Plant Pathology NC State University Raleigh North Carolina USA; ^2^ Fiber and Biopolymer Research Institute (FBRI) Department of Plant and Soil Science Texas Tech University Texas USA; ^3^ Plant Science Research Unit USDA‐ARS Raleigh North Carolina USA

**Keywords:** hypersensitive response, maize, VIGS

## Abstract

Plant disease resistance proteins (R‐proteins) detect specific pathogen‐derived molecules, triggering a defence response often including a rapid localized cell death at the point of pathogen penetration called the hypersensitive response (HR). The maize *Rp1‐D21* gene encodes a protein that triggers a spontaneous HR causing spots on leaves in the absence of any pathogen. Previously, we used fine mapping and functional analysis in a *Nicotiana benthamiana* transient expression system to identify and characterize a number of genes associated with variation in *Rp1‐D21*‐induced HR. Here we describe a system for characterizing genes mediating HR, using virus‐induced gene silencing (VIGS) in a maize line carrying *Rp1‐D21*. We assess the roles of 12 candidate genes. Three of these genes, *SGT1*, *RAR1*, and *HSP90*, are required for HR induced by a number of R‐proteins across several plant–pathogen systems. We confirmed that maize HSP90 was required for full *Rp1‐D21*‐induced HR. However, suppression of SGT1 expression unexpectedly increased the severity of *Rp1‐D21*‐induced HR while suppression of RAR1 expression had no measurable effect. We confirmed the effects on HR of two genes we had previously validated in the *N. benthamiana* system, *hydroxycinnamoyltransferase* and *caffeoyl CoA O‐methyltransferase*. We further showed the suppression the expression of two previously uncharacterized, candidate genes, *IQ calmodulin binding protein* (*IQM3*) and *vacuolar protein sorting protein 37*, suppressed *Rp1‐D21*‐induced HR. This approach is an efficient way to characterize the roles of genes modulating the hypersensitive defence response and other dominant lesion phenotypes in maize.

## Graphical abstract

In this study we used a virus‐induced gene silencing technique to characterize the roles of 12 genes in controlling the hypersensitive cell death defence response in maize.

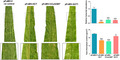

## 1 INTRODUCTION

The mechanisms underlying programmed cell death (PCD) in plants are complex and include several interacting pathways involved in plant development and disease resistance (Kuriyama and Fukuda, 2002; Williams and Dickman, 2008; Reape et al., 2015; Bruggeman et al., 2015; Dickman et al., 2017). The hypersensitive response (HR) is a specific form of PCD that is characterized by rapid, localized cell death at a point of pathogen infiltration or damage (Wu and Baldwin, 2010; Coll et al., 2011; Balint‐Kurti, 2019). It is mediated by dominant resistance (R‐) proteins, which are activated in response to the detection of specific pathogen‐derived effector proteins (Bent and Mackey, 2007). This activation leads to a so‐called effector‐triggered immunity (ETI) response that often, though not always (Künstler et al., 2016; Laflamme et al., 2020), includes HR. Other than HR, the ETI response is characterized by several physiological changes, including cell wall strengthening, ion fluxes, changes in phenylpropanoid biosynthesis, and oxidative burst (Kosack and Jones, 1996). Because inappropriate activation of HR is potentially devastating for the host, R‐proteins must be tightly controlled so that in the absence of the pathogen they remain in an inactive form that can be rapidly activated when the presence of the cognate pathogen effector is detected (Balint‐Kurti, 2019; Wang et al., 2019). Pathogens can also be recognized through detection of conserved microbe‐associated molecular patterns (MAMPs) by surface‐located receptors called pattern recognition receptors (PRRs). MAMP recognition leads to activation of the MAMP‐triggered immunity (MTI) response, which is qualitatively similar to ETI though quantitatively lower, usually not involving an HR (Newman et al., 2013). Recent studies suggest that a functional MTI response is important to enable a full ETI response (Ngou et al., 2020; Yuan et al., 2020).

Most R‐proteins possess nucleotide‐binding site motifs and leucine‐rich repeat domains and are consequently known as NLRs. Within this class of proteins, two major groups exist, defined by whether they possess a coiled‐coil (CC) domain or a Toll/interleukin‐1 receptor (TIR) domain at their N‐terminus. The *Rp1* locus in maize is a complex locus encoding multiple tandemly repeated NLR paralogs, several of which confer resistance to specific races of *Puccinia sorghi*, the causal agent of maize common rust (Hulbert, 1997). Unequal crossover events at this locus are relatively frequent (Hulbert and Bennetzen, 1991). The *Rp1‐D21* allele is the product of an intragenic recombination event between two *Rp1* paralogs, *Rp1‐D* and *Rp1‐dp2*. It encodes a protein that can be spontaneously activated without pathogen recognition and hence causes HR lesions on the leaves of maize plants in the absence of pathogen (Hu et al., 1996; Collins et al., 1999; Smith et al., 2010; Wang et al., 2015b). The severity of the HR conferred by *Rp1‐D21* is modulated by genetic background as well as by various environmental factors (Hu et al., 1996; Smith et al., 2010; Chaikam et al., 2011; Negeri et al., 2013).

We identified 44 loci associated with variation in the *Rp1‐D21*‐induced HR phenotype using genome‐wide association (GWA) analysis in the maize nested association mapping (NAM) population (Olukolu et al., 2013). The NAM population is a particularly powerful mapping resource because it is large (5,000 lines), captures a large number of recombination events, and is very densely genotyped (Yu et al., 2008; McMullen et al., 2009). This means that quantitative trait loci (QTLs) can be mapped with very high precision, often to single‐gene resolution. We were able to identify candidate causal genes at many of the 44 loci and, in several cases, have verified their effects on HR caused by *Rp1‐D21* using techniques including transient coexpression in *Nicotiana benthamiana*, physical association assays including coimmunoprecipitation and bimolecular fluorescence complementation (BiFC) with *Rp1‐D21* and, in one case, the analysis of a maize line in which the gene was overexpressed due to a nearby transposon insertion (Wang et al., 2015a; Wang and Balint‐Kurti, 2016; He et al., 2019a,2019b). In many cases it is likely that the genes modulating *Rp1‐D21*‐mediated HR are also involved in modulating the wild‐type defence response. Using the phenotype conferred by *Rp1‐D21* as a reporter has made the identification of these important components of the defence response more straightforward. One of the main weaknesses of these studies was that the main validating evidence was generated in an ectopic system using agroinfiltration of *N. benthamiana* because we did not have the ability to easily modulate gene expression in maize.

In many respects maize is an ideal system for genetic research, with abundant publicly available experimental resources (Nannas and Dawe, 2015). The one area where the maize system is less tractable than other plant model genetic systems such as *Arabidopsis*, rice, and tomato is reverse genetics. With the burgeoning resources for maize genome mapping and analysis, the identification of candidate genes underlying traits of interest is becoming easier and more accurate, exacerbating the bottleneck created by the lack of resources for reverse genetics.

Reverse genetics resources are more readily available in several other plant genetic systems. Whereas *Arabidopsis*, tomato, and rice are relatively easily transformable in any laboratory skilled in tissue culture and molecular biology, maize transformation requires specialized explants and tissue culture techniques, and can only be achieved in certain genetic backgrounds (Yadava et al., 2017). As such, maize transformation is largely limited to a few service laboratories in the public sector, which are necessarily rather costly and can take up to 4–6 months to produce a transgenic line. It should be noted that new technologies promise to improve this situation (Jones et al., 2019), but they are not yet in the public domain. The recent availability of CRISPR/Cas9 technology in maize is revolutionizing the field by allowing us to make targeted genome edits (Svitashev et al., 2016; Liu et al., 2020) but is still limited by a relatively inefficient transformation system.

Populations of sequence‐indexed mutant maize lines also exist, the most extensive and available of which is the UniformMu population, which consists of a population of 14,024 stocks in a uniform background of the W22 maize line, each of which carries a number of unique, stabilized transpositions of the *Mu* transposable element. The transposition sites have been sequenced and together the population carries *Mu* insertions in or near c.42% of maize genes. However, only about half of these are in the protein coding sequences (McCarty et al., 2018) meaning that loss‐of‐function alleles for most maize genes are not available.

In this study, we report the use of a recently developed system for virus‐induced gene silencing (VIGS) in maize, based on foxtail mosaic virus (FoMV) (Mei et al., 2016; Mei and Whitham, 2018), to characterize genes associated with *Rp1‐D21* activity. We developed a quantitative screen for alteration of *Rp1‐D21*‐induced HR and used it to characterize the effects of 12 genes. These included the four genes whose effects we had previously validated using the *N. benthamiana* system (*HCT*, *CCoAOMT*, *QCR7*, and *PGH1*), other candidates previously identified from GWA analysis (*IQM3*, *VPS37*, *Pk1b*, *SL11*, *LOX9*), and several genes previously shown to modulate NLRs in other systems (*SGT1*, *HSP90*, *RAR1*). The FoMV‐VIGS system produced robust evidence that validated some of our previous work and further elucidated the mechanisms that control HR in maize.

## 2 RESULTS AND DISCUSSION

### 2.1 Reduction of *Rp1‐D21* transcript levels using foxtail mosaic virus‐mediated VIGS

The maize *Rp1‐D21* protein is a chimeric autoactive NLR that triggers spontaneous HR in the absence of a pathogen. A previous genome‐wide association study (GWAS) analysis in maize precisely mapped 44 loci associated with variation in *Rp1‐D21*‐induced HR and tentatively identified candidate genes underlying these effects (Olukolu et al., 2014). Follow‐up studies validated the roles of two of the candidate genes encoding the enzymes HCT and CCoAOMT, which catalyse consecutive steps in the lignin biosynthesis pathway (Wang et al., 2015a; Wang and Balint‐Kurti, 2016). The most important aspect of these studies was the observation that specific members of the maize *HCT* and *CCoAOMT* gene families (Table 1) suppressed *Rp1‐D21*‐induced HR in *N. benthamiana* transient expression assays. Using similar approaches we subsequently validated the effects on *Rp1‐D21* activity of two further candidate genes identified in the original GWAS study: a polygalacturanase, *PGH1* (He et al., 2019a), and a gene predicted to encode a small protein, QCR7, forming part of the cytochrome b‐c1 complex (He et al., 2019b). These studies were performed in an ectopic system because we lacked the ability to conveniently manipulate gene activity in maize.

**TABLE 1:** List of candidate genes associated with variation in the severity of *Rp1‐D21*‐induced hypersensitive response (HR)

Gene ID	Annotation	Silencing level (fold)	Effect on *Rp1‐D21*	References
*Rp1‐D21*‐middle	*Rp1‐D21*	3.14	Suppress	Olukolu et al. (2014); Wang et al. (2015)
GRMZM2G012631^a^	*HSP90*	16.83	Suppress	
GRMZM2G017616	*Lipoxygenase9* (*LOX9*)	NS	–	Olukolu et al. (2014)
GRMZM2G023575	*Modifier of rudimentary protein* (*VPS37*)	1.63	Suppress	Olukolu et al. (2014)
GRMZM2G061806	*Hydroxycinnamoyl‐CoA shikimate/quinate* (*HCT*)	10.52	Enhance	Olukolu et al. (2014); Wang et al. (2015)
GRMZM2G099363	*Caffeoyl‐CoA O‐methyltransferase* (*CCoAOMT*)	4.16	Enhance	Olukolu et al. (2014); Wang and Balint‐Kurti (2016)
GRMZM2G105019^b^	*SGT1*	4.61	Enhance	
GRMZM2G135763	*Polygalacturonase* (*PGH1*)	2.46	None	Olukolu et al. (2014); He et al. (2019)^a^
GRMZM2G144042	*Protein kinase 1b* (*Pk1b*)	NS	–	Olukolu et al. (2014)
GRMZM2G318346	*Cytochrome bd ubiquinol oxidase* (*QCR7*)	2.08	None	Olukolu et al. (2014); He et al. (2019)^b^
GRMZM2G351387	*Spotted leaf 11*/*plant U‐box 13* (*SL11*)	NS	–	Olukolu et al. (2014)
GRMZM2G439311^c^	*IQ calmodulin‐binding motif family protein* (*IQM3*)	7.87	Suppress	Olukolu et al. (2014)
GRMZM5G868908	*RAR1*	3.93	None	

NS, not significant.

^a^ The PCR product used for virus‐induced gene silencing (VIGS) was derived from GRMZM2G012631 but the resulting construct could additionally target four other homologs in the genome.

^b^ The PCR product used for VIGS was derived from GRMZM2G105019 but the resulting construct could target two homologs in the genome.

^c^ Gene annotation is not correct in B73 genome v 4. We designed the primers based on our sequence from amplified PCR product.

A maize VIGS system, based on FoMV, was recently shown to be capable of efficiently suppressing the transcript level of specific genes of interest like *phytoene desaturase* (*PDS*), which causes photobleaching of leaves (Mei et al., 2016). This system was significantly more effective than the previously reported maize VIGS system based on brome mosaic virus (Ding et al., 2006; Benavente et al., 2012). We used this system for the further characterization of genes associated with the modification of the HR phenotype conferred by *Rp1‐D21*.

**FIGURE 1:**
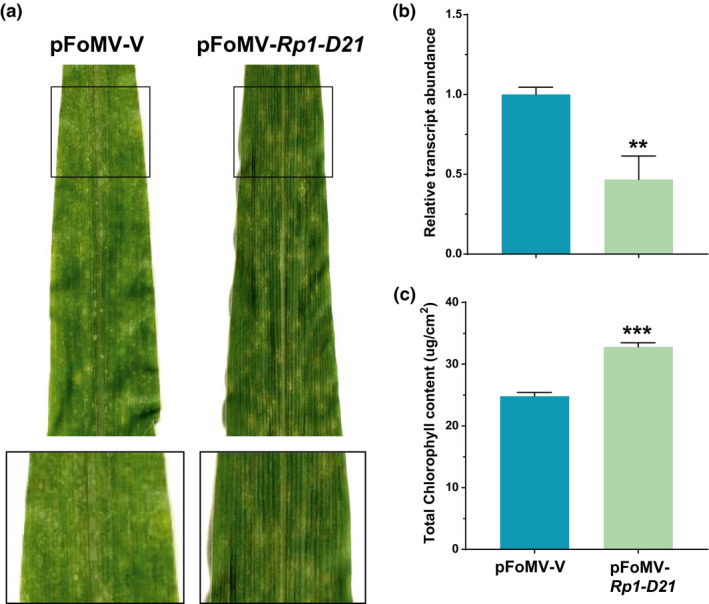
Infection of B73:*Rp1‐D21* by pFoMV‐V and pFoMV‐*Rp1‐D21*. (a) Image of fourth leaves from inoculated plants 14 days postinoculation (dpi). Black squares outline the area that is shown magnified directly below the main picture. (b) Real‐time quantitative reverse transcription PCR analysis of *Rp1‐D21* expression in B73:*Rp1‐D21*. Significant suppression of *Rp1‐D21* transcripts is detected in the fourth leaves of plants infected with pFoMV‐*Rp1‐D21* compared with those infected with pFoMV‐V. Data normalized using *actin* as a reference. (c) Soil plant analysis development data from inoculated plants. Significant increase of chlorophyll content is observed in the fourth leaves of B73:*Rp1‐D21* plants infected with pFoMV‐*Rp1‐D21* compared with the empty vector. (**p < .01, ***p < .001). Value is derived from at least three biological replications

First, we used the FoMV VIGS system to silence *Rp1‐D21* itself in B73:*Rp1‐D21*. A 469‐bp fragment corresponding to the leucine‐rich repeat (LRR) region of *Rp1‐D21* was inserted in the antisense orientation at the *Xba*I and *Xho*I cloning sites of pFoMV‐V (Table 1) to create the pFoMV‐*Rp1‐D21* construct designed to suppress expression of *Rp1‐D21*. Lesions caused by spontaneous HR were visible on the first and second leaves of B73:*Rp1‐D21* plants c.10 days after germination. Significant reduction of HR in B73:*Rp1‐D21* plants was observed at 14 days after infection with FoMV‐*Rp1‐D21*, compared to B73:*Rp1‐D21* infected with pFoMV‐V (the empty vector control; Figure 1).

*Rp1‐D21* transcript levels in B73:*Rp1‐D21* plants infected with FoMV‐*Rp1‐D21* were 2.14‐fold lower than control plants infected by FoMV‐V (Figure 1b). As well as causing necrotic spots, the presence of *Rp1‐D21* also causes a general leaf chlorosis. In addition to visual assessment, the severity of the *Rp1‐D21* phenotype was, therefore, also measured using a soil plant analysis development (SPAD) meter to assess total leaf chlorophyll content as a proxy. The leaves of B73:*Rp1‐D21* plants infected with pFoMV‐*Rp1‐D21* possessed higher chlorophyll content than those infected with pFoMV‐V, confirming our visual assessment that the level of HR had been reduced (Figure 1c).

B73 plants (i.e., plants that did not carry *Rp1‐D21*) infected by pFoMV‐*Rp1‐D21* or pFoMV‐V developed typical viral symptoms and did not show any HR symptoms, as expected. Representative pictures of B73 plants infected with pFoMV‐*Rp1‐D21* and every other VIGS construct used in this study are shown in Figure S1. pFoMV‐*PDS* constructs designed to suppress the expression of the *PDS* gene were also used as a way to test the system. B73:*Rp1‐D21* plants infected with pFoMV‐*PDS* showed bleached “stripes” typical of the phenotype expected from suppression of *PDS* expression and *PDS* transcript levels were reduced by 3.14‐fold compared to B73:*Rp1‐D21* plants infected with pFoMV‐V. Chlorophyll content was also slightly lower in B73:*Rp1‐D21* plants infected with FoMV‐*PDS* compared to those infected with pFoMV‐V (Figure S2).

These results demonstrated that the FoMV VIGS system worked well in our hands and could be used as a tool to interrogate the mechanisms controlling the expression of HR induced by *Rp1‐D21*. Below we describe our findings using the FoMV VIGS system to suppress expression of 12 genes in a B73:*Rp1‐D21* background. These genes were identified as candidate genes for HR modulation either from our previous work or from the general literature. We first describe the six genes whose suppression led to changes in levels of HR (three up, three down), then the three whose suppression had no measurable effect, and finally three genes whose expression we were unable to suppress with this system.

### 2.2 Silencing of *HCT*, *CCoAOMT*, and *Sgt1* enhances *Rp1‐D21*‐induced HR

#### 2.2.1 HCT *and* CCoAOMT

*Hydroxycinnamoyltransferase* (*HCT*) and *caffeoyl CoA O‐methyltransferase* (*CCoAOMT*) (Ye et al., 1994; Hoffmann et al., 2004) are both present as gene families in maize and catalyse adjacent steps in the lignin biosynthetic pathway. We previously demonstrated that members of each gene family (the closely linked *HCT* genes *GRMZM2G061806* and *GRMZM2G114918*, referred to as *HCT1806* and *HCT4918*, respectively, and the *CCoAOMT2* gene *GRMZM2G099363*) were present at loci associated with modulation of HR conferred by *Rp1‐D21* and were able to suppress *Rp1‐D21*‐induced cell death when coexpressed with *Rp1‐D21* in *N. benthamiana* (Wang et al., 2015a; Wang and Balint‐Kurti, 2016). We also demonstrated that HCT1806, HCT4918, CCoAOMT2, and *Rp1‐D21* proteins physically interacted in vitro.

VIGS constructs pFoMV*‐HCT* and pFoMV*‐CCoAOMT*, designed to silence *HCT1806*/*HCT4918* and *CCoAOMT2*, respectively, were constructed in pFoMV‐V (Table 1). In the case of pFoMV*‐HCT*, the construct was able to silence both *HCT1806* and *HCT4918* due to their high homology (88%). The primers used for quantitative reverse transcription PCR (RT‐qPCR) were also designed to amplify transcripts from both genes. In the case of pFoMV‐*CCoAOMT* the construct was predicted to silence both the *CCoAOMT2* gene *GRMZM2G099363* and a closely related gene, *GRMZM2G127948*; however, the primers used for RT‐qPCR were also designed to specifically amplify cDNA derived from *GRMZM2G099363*.

**FIGURE 2:**
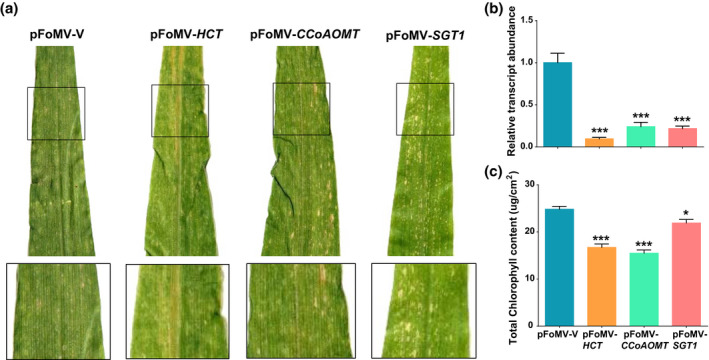
Increased hypersensitive response (HR) cell death in B73:*Rp1‐D21* plants infected with pFoMV‐*HCT*, pFoMV‐*CCoAOMT*, and pFoMV‐*SGT1* compared with those infected with pFoMV‐V. (a) Image of the fourth leaves from inoculated plants 14 days postinoculation (dpi.) Black squares outline area that is shown magnified directly below the main picture. (b) Real‐time quantitative reverse transcription PCR analysis of *HCT*, *CCoAOMT*, and *SGT1:* significant suppression of *HCT, CCoAOMT*, and *SGT1* transcripts were detected in the fourth leaves of plants that were infected with each virus‐induced gene silencing construct relative to pFoMV‐V. Values were normalized using *actin* as a reference. (c) Soil plant analysis development data from inoculated plants. Significant suppression of chlorophyll content was observed in the fourth leaves of plants infected with each virus‐induced gene silencing construct relative to pFoMV‐V. (*p < .05, **p < .01, ***p < .001). Values are derived from at least three biological replications

The transcript levels of *HCT1806*/*HCT4918* and *CCoAOMT2* in B73:*Rp1‐D21* plants infected with pFoMV*‐HCT* and pFoMV*‐CCoAOMT*, respectively, were reduced by 10.52‐ and 4.16‐fold compared to plants infected with pFoMV‐V 14 days after viral infection (Figure 2b). Stronger HR was observed in B73:*Rp1‐D21* lines infected with pFoMV*‐HCT* or pFoMV*‐CCoAOMT* compared to B73:*Rp1‐D21* lines carrying pFoMV*‐*V as determined visually (Figure 2a) and by SPAD analysis (Figure 2c).

The enhancement of HR induced by *Rp1‐D21* associated with suppression of the *HCT* and *CCoAOMT* genes in maize was consistent with our previous findings that their overexpression in *N. benthamiana* suppressed HR conferred by *Rp1‐D21* (Wang et al., 2015a; Wang and Balint‐Kurti, 2016). These findings suggested that the FoMV VIGS system was indeed a promising tool to investigate the mechanisms of HR modulation and encouraged us to investigate other genes in a similar way.

#### 2.2.2 SGT1

The SUPPRESSOR OF THE G2 ALLELE OF SKP1 (SGT1), HEAT SHOCK PROTEIN90 (HSP90), and REQUIRED FOR MLA12 RESISTANCE (RAR1) proteins form a molecular chaperone complex that is an important signalling component of plant immune responses (Shirasu, 2009; Zhang et al., 2010b). This complex interacts with NLRs in numerous systems, stabilizing them and allowing their proper maturation and function (Zhang et al., 2010b). Within this complex, SGT1 plays a central role as it recruits the NLR and also interacts with the E3 ubiquitin ligase subunit Skp1 (Catlett and Kaplan, 2006), part of the Skp1/Cullin/F‐box (SCF) ubiquitin ligase complex that targets proteins for degradation (Azevedo et al., 2006; Cheng et al., 2011; Gou et al., 2015). Suppression or mutation of any one of RAR1, SGT1, or HSP90 in many interactions is sufficient to abolish HR and to reduce NLR protein levels (e.g., Hubert et al., 2003; Bieri et al., 2004; Scofield et al., 2005; Azevedo et al., 2006; Kim et al., 2018).

The two maize homologs of *SGT1* annotated in maize (B73 genome v. 3) are *GRMZM2G105019* and *GRMZM2G149704* (here called *SGT5019* and *SGT9704*, respectively). They share a nucleotide identity of 93%. It was not possible to silence only one of these genes specifically due to their high identity across the full length of their coding sequences, so we designed pFoMV‐*SGT1* to suppress both variants of *SGT1*. Likewise, the primers used for RT‐qPCR were designed to detect the transcripts from both *SGT1* homologs. Infection of B73:*Rp1‐D21* plants with pFoMV‐*SGT1* reduced total *SGT1* transcript levels by 4.61‐fold compared to control plants (Figure 2b) and enhanced *Rp1‐D21*‐associated HR (Figure 2a,c). We also noted that pFoMV‐*SGT1* infection of B73 plants often caused the development of lesions that were not seen when B73 was infected with pFoMV‐V or with the other constructs used in this study (Figure S1), suggesting that perhaps suppression of *SGT1* expression was also causing spontaneous HR in the absence of *Rp1‐D21*.

Suppression of *SGT1* expression in most systems studied resulted in lower accumulation of the activated NLR protein and the consequent suppression of resistance and HR (Holt et al., 2005; Cheng et al., 2011). The enhancement of HR associated with suppression of *SGT1* expression in maize that we observed was therefore unexpected. However, the role of SGT1 is not completely understood. While it is required for HR induced by NLRs in most studied cases, there are several reports of SGT proteins playing an HR‐inhibitory role. In *Arabidopsis*, inactivation of one of the two functional *SGT1* genes led to increased accumulation of the RPS5 NBS‐LRR R‐protein, suggesting that SGT1 antagonized the effect of RAR1 (Holt et al., 2005), acting to reduce levels of NLR proteins. Similarly, Gou et al. (2015) showed that overexpression of *SGT1b* suppressed SNC1 protein accumulation, leading to suppression of SNC1‐triggered cell death in *Arabidopsis*. The role of SGT1 may vary depending on the species, the specific member of the gene family in the cases where multiple *SGT1* genes are present in the genome, and the particular NLR or its activation state (Azevedo et al., 2006).

**FIGURE 3:**
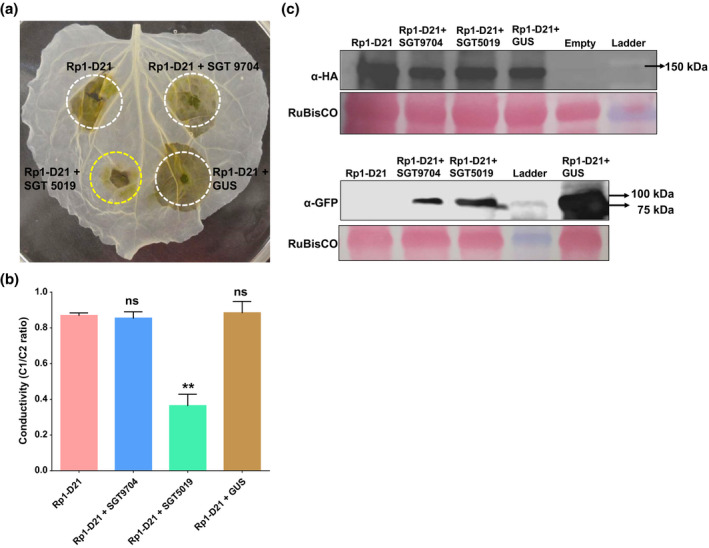
Suppression of *Rp1‐D21*‐induced cell death by coexpression of *SGT15019* but not *SGT9704* in *Nicotiana benthamiana*. (a) Transient coexpression of *SGT5019* or *SGT9704* with *Rp1‐D21* in *N. benthamiana*. β‐glucuronidase (*GUS*) was used as a negative control. *Agrobacterium* carrying each construct was diluted to a final concentration of OD_600_ = 1.0. Infiltrated regions are marked with ovals. (b) Ion leakage conductivity was measured at 36 hr after coexpression of *SGT5019*, *SGT9704*, or *GUS* with *Rp1‐D21*. Data represent means ± *SD* from three independent plants. (*significant difference from *Rp1‐D21* positive control at *p* < .01; ns, not significant). (c) Total protein extracted from agroinfiltrated leaves at 30 hr postinoculation (hpi). Leaves infiltrated with empty vector were used as a negative control. Anti‐HA was used to detect *Rp1‐D21* and anti‐GFP was used to detect SGT5019, SGT9704, or GUS. Equal loading of protein samples is shown by Ponceau S staining of RuBisCO band. Each experiment was performed with three replications

Because the effects of SGT5019 and SGT9704 could not be differentiated using VIGS we turned to the *N. benthamiana* transient expression system. We coexpressed each *SGT1* gene with *Rp1‐D21* in tobacco leaves. Coexpression of *SGT5019* but not *SGT9704* with *Rp1‐D21* suppressed *Rp1‐D21*‐mediated HR (Figure 3a). Ion leakage conductivity data confirmed that coexpression of *Rp1‐D21* with *SGT5019* significantly reduced ion leakage compared with coexpression of a GUS:GFP control (Figure 3b). This indicates that SGT5019 is involved in regulating *Rp1‐D21*‐induced cell death. Based on this result we speculated that SGT5019 might suppress *Rp1‐D21*‐induced cell death by reducing *Rp1‐D21* protein accumulation. To evaluate this possibility, the protein levels of *Rp1‐D21* and SGT1 were quantified by western blot. *Rp1‐D21* was detected using antibodies against an HA epitope tag that had been added to the C‐terminus of *Rp1‐D21*. SGT1 homologs and β‐glucuronidase (GUS) were detected using antibodies that detected their attached green fluorescent protein (GFP) epitope tags. *Rp1‐D21* was detected at similar levels when coexpressed with GUS:GFP, SGT9704, and SGT5019 (Figure 3c), and the levels of the two SGT1 proteins themselves were comparable. This indicated that there was no evidence that the suppressive effect of SGT5019 on HR induced by *Rp1‐D21* was due to reduced *Rp1‐D21* protein levels.

### 2.3 Silencing of *IQM3*, *VPS37*, and *HSP90* suppresses *Rp1‐D21*‐induced cell death

#### 2.3.1 IQM3

*GRMZM2G439311* encodes IQ calmodulin‐binding (IQM3) protein and is less than 4 kb from a single nucleotide polymorphism (SNP) positioned on chromosome 4 at 188,208,471 bp that was highly associated with variation in *Rp1‐D21*‐induced HR (Olukolu et al., 2014). The IQ motif (IQXXXRGXXXR) is a common motif that binds calmodulin, an important calcium sensor that regulates many aspects of the cytoskeleton and mitosis and many other cell functions (Bähler and Rhoads, 2002). Several studies have shown the importance of calmodulin and calmodulin‐like proteins in regulating plant defence and HR. *CaCaM1* (*pepper calmodulin1*) overexpression conferred disease resistance by regulating reactive oxygen species accumulation in pepper (Choi et al., 2009). A point mutation in *CML24* (*Calmodulin 24*) gene impaired HR against avirulent *Pseudomonas syringae* pv*. tomato* (*avrRpt2*) in *Arabidopsis thaliana* and HR was accelerated against *avrRpt2* by overexpressing *CML43* (*Calmodulin 43*) (Chiasson et al., 2005; Ma et al., 2008). Based on these studies, we postulated *IQM3* may have some role in regulating *Rp1‐D21* mediated cell death and developed the pFoMV‐*IQM3* to suppress *IQM3* expression in maize.

**FIGURE 4:**
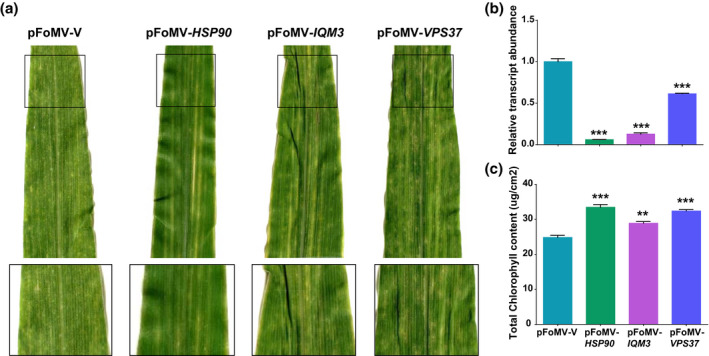
Suppression of hypersensitive response (HR) in B73:*Rp1‐D21* plants infected with pFoMV‐*HSP90*, pFoMV‐*IQM3*, and pFoMV‐*VPS37*. (a) Image of the fourth leaves from inoculated plants 14 days postinoculation (dpi). Black squares outline area that is shown magnified directly below the main picture. (b) Real‐time quantitative reverse transcription PCR analysis of *HSP90*, *IQM3*, and *VPS37* in B73:*Rp1‐D21*. Significant suppression of *HSP90, IQM3*, and *VPS37* transcripts was detected in the fourth leaves of plants infected with each virus‐induced gene silencing construct relative to pFoMV‐V. Values normalized using *actin* as a reference. (c) Soil plant analysis development data from inoculated plants. Significant suppression of chlorophyll content was observed in the fourth leaves of plants that were infected with each virus‐induced gene silencing construct relative to pFoMV‐V. (*p < .05, **p < .01, ***p < .001). Values are derived from at least three biological replications

The transcript abundance of *IQM3* was 7.87‐fold lower in pFoMV‐*IQM3*‐infected plants compared to pFoMV‐V‐infected plants (Figure 4b). Phenotypic observations and chlorophyll data showed that B73:*Rp1‐D21* plants infected with pFoMV‐*IQM3* had less severe HR lesions than B73:*Rp1‐D21* plants infected with pFoMV‐V (Figure 4a,c). These results suggest that *IQM3* silencing suppresses *Rp1‐D21*‐induced cell death, and therefore that this protein enhances HR induced by *Rp1‐D21*.

#### 2.3.2 VPS37

*GRMZM2G023575* encodes the vacuolar protein sorting protein 37 (VPS37). It is situated 28.1 kb from a SNP on chromosome 1 at 18,100,677 bp, which is significantly associated with variation in *Rp1‐D21*‐induced HR (Olukolu et al., 2014). VPS37 is a component of the ESCRT‐I (Endosomal Sorting Complex Required for Transport) complex, which together with the ESCRT‐0, ‐I, ‐II and ‐III complexes and the Vps4 complex, as well as other components, enable membrane remodelling and the processing of ubiquitinated proteins to the lysosome (Schmidt and Teis, 2012). In *Arabidopsis* the ESCRT system and VPS37 in particular have been shown to be important for the endocytic transport of the plant immune receptor FLS2, an LRR‐receptor kinase PRR, at the plasma membrane after activation triggered by bacterial flagellin (Spallek et al., 2013). VPS37‐1 colocalized with FLS2 at endosome upon flagellin elicitation and *vps37‐1* mutants compromised vacuolar sorting of FLS2 and flagellin‐triggered stomatal closure, and displayed impaired immunity to the bacterial pathogen *P. syringae* and to the biotrophic oomycete *Hyaloperonospora arabidopsidis* (Lu et al., 2012). While FLS2 activation and the resulting MTI do not generally lead to an HR, a strong connection has recently been shown between a functional MTI response and a full ETI response, including HR (Ngou et al., 2020; Yuan et al., 2020).

Silencing of *VPS37* suppressed *Rp1‐D21*‐induced HR in maize (Figure 4a,c). The *VPS37* transcript level was reduced by 1.63‐fold in plants infected with pFoMV‐*VPS37* compared to control plants infected with pFoMV‐V (Figure 4b) and pFoMV‐*VPS37*‐infected leaves had higher chlorophyll content than those infected by FoMV‐V (Figure 4c). This suggests that the ESCRT endosomal sorting complex might be important for controlling the *Rp1‐D21*‐mediated defence response.

#### 2.3.3 HSP90

HSP90 is a component of the SGT1/HSP90/RAR1 chaperone complex that has been implicated in facilitating NLR‐mediated HR in a number of systems and is described above. HSP90 is a protein chaperone that can stabilize NLR proteins or recruit cofactors that allow NLR proteins to function properly in plant defence signalling. In previous studies *HSP90*‐silenced plants exhibited growth reduction and yellowing leaves as well as suppressed NLR‐mediated HR and resistance to pathogens (Liu et al., 2004; Zhang et al., 2004; Scofield et al., 2005; Bos et al., 2006; Cakir et al., 2010; Shibata et al., 2011; Kim et al., 2018). We wanted to determine if HSP90 also functions in *Rp1‐D21*‐induced HR in maize.

There are five HSP90 homologs in the maize genome (*GRMZM2G024668, GRMZM2G012631, GRMZM2G112165, GRMZM2G069651, GRMZM5G833699*). Nucleotide identities between them are 82%–93% and pFoMV‐*HSP90* was designed to knock down all five gene variant transcripts via cross‐complementarity to mature mRNA exons. Similarly, the primers used for RT‐qPCR were designed to amplify transcripts from all five genes. We observed that cell death was markedly reduced in B73:*Rp1‐D21* infected with pFoMV‐*HSP90* (Figure 4a) compared to B73:*Rp1‐D21* infected with pFoMV‐V. The overall transcript levels of the five *HSP90* homologs in the silenced plants was 16.83‐fold lower than in control plants (Figure 4b). Total chlorophyll content was elevated compared to the plants infected by FoMV‐V. In addition, both B73:*Rp1‐D21* and B73 infected with FoMV‐*HSP90* displayed yellow stripes and noticeably reduced growth (Figures 4a and S1). Somewhat similar results were reported in *N. benthamiana*, where suppression of *HSP90* expression led to stunting and eventual death (Liu et al., 2004). While we were not able to differentiate the roles of the five *HSP90* homologs, these results suggest that HSP90 may play a similar role in controlling HR associated with *Rp1‐D21* in maize as it does in most of the other systems in which it has been investigated.

### 2.4 Silencing of *RAR1*, *PGH1*, and *QCR7* do not alter the *Rp1‐D21*‐induced HR

#### 2.4.1 RAR1

**FIGURE 5:**
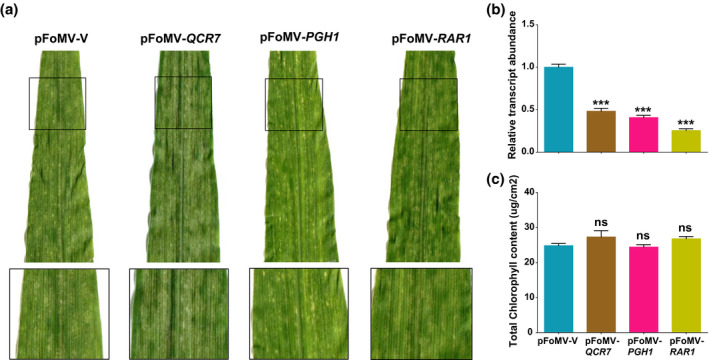
Phenotypes of B73:*Rp1‐D21* plants infected with pFoMV‐*QCR7*, pFoMV‐*PGH1*, and pFoMV‐*RAR1*. (a) Image of the fourth leaves from inoculated plants 14 days postinoculation (dpi). Black squares outline the area that is shown magnified directly below the main picture. (b) Real‐time quantitative reverse transcription PCR analysis of *QCR7*, *PGH1*, and *RAR1* in B73:*Rp1‐D21*. Significant suppression of *QCR7, PGH1*, and *RAR1* transcripts was detected in systemic leaves of plants infected with each virus‐induced gene silencing construct relative to pFoMV‐V. Values normalized using *actin* as a reference. (c) Soil plant analysis development data from inoculated plants. No significant difference in chlorophyll content was observed in the fourth leaves of plants that were infected with each virus‐induced gene silencing construct relative to pFoMV‐V. ns, not significant. Values are derived from at least three biological replications

RAR1 is the third member of the SGT1/HSP90/RAR1 chaperone complex described above. However, *RAR1*‐silenced plants do not always exhibit reduced NLR‐mediated resistance (Shibata et al., 2011; Kim et al., 2018). Three RAR1 homologs exist in the maize genome (*GRMZM5G868908, GRMZM2G156105* and *GRMZM5G881347*). *GRMZM5G868908* and *GRMZM2G156105* are 93% identical at the nucleotide level over the whole length of their 587 bp cDNA, while *GRMZM5G881347* appears to be an incomplete form of RAR1, with several deletions. pFoMV‐*RAR1* was designed to silence both *GRMZM5G868908* and *GRMZM2G156105* but not *GRMZM5G881347*. Similarly, the primers used for RT‐qPCR were designed to amplify transcripts from *GRMZM5G868908* and *GRMZM2G156105* but not *GRMZM5G881347*. No phenotypic differences associated with silencing were observed in plants infected with FoMV‐*RAR1*. *Rp1‐D21*‐induced cell death was unchanged despite a 3.93‐fold decrease in the *RAR1* transcripts measured as compared to FoMV‐V (Figure 5). These data do not support a role for RAR1 in the *Rp1‐D21*‐induced HR.

#### 2.4.2 ZmPGH1 *and* ZmQCR7

*ZmPGH1* (*Zea mays polygalacturonase homolog 1 GRMZM2G135763*) was identified from the prior GWAS (Olukolu et al., 2014) as a candidate gene for modulation of *Rp1‐D21*‐mediated HR. In subsequent work, He et al. (2019a) showed that coexpression of *ZmPGH1* inhibited HR induced by *Rp1‐D21* and by another autoactive NLR RPM1(D505V) in *N. benthamiana*. We therefore expected that *ZmPGH1* suppression might enhance the *Rp1‐D21*‐induced HR. However, reduction of the *PGH1* transcript level by 2.46‐fold compared to control had no obvious phenotypic effect on *Rp1‐D21*‐induced HR in B73:*Rp1‐D21* (Figure 5).

Similarly to *ZmPGH1*, *ZmQCR7* (*GRMZM2G318346*, *cytochrome b–c1 complex subunit 7*) was also identified from the prior GWAS (Olukolu et al., 2014) as a candidate gene for modulation of *Rp1‐D21*‐mediated HR. Also similar to *ZmPGH1,* subsequent analysis showed that coexpression of *ZmPGH1* inhibited HR induced by *Rp1‐D21* and RPM1(D505V) (He et al., 2019b). Silencing of *QCR7* by FoMV‐*QCR7* displayed no obvious effect on the *Rp1‐D21*‐induced HR in B73:*Rp1‐D21*, although the *QCR7* transcript level was reduced by 2.08‐fold compared to control plants (Figure 5).

Based on previous results, we anticipated that suppressing the expression of *ZmPGH1* and *ZmQCR7* in B73:*Rp1‐D21* plants would enhance *Rp1‐D21*‐induced HR. The fact that we did not observe any such enhancement may be due to several factors. First, the level of gene suppression may not have been sufficient. *ZmPGH1* and *ZmQCR7* transcript levels were suppressed 2.46‐ and 2.08‐fold, respectively. By contrast, the *CCoAOMT2* and *HCT* genes were suppressed 10.52‐ and 4.16‐fold, respectively. This means that substantial amounts of transcript were still available for translation. Furthermore, relationships between mRNA levels, protein levels, and protein activity are nonlinear and unpredictable (Walley et al., 2016). Both genes are members of broader gene families; however, functional redundancy between family members seems unlikely to be the cause of the lack of effect because assuming these genes are indeed the causal genes underlying the original GWAS association (Olukolu et al., 2014), functional redundancy would have also precluded identifying the associations in the first place. It should also be noted that the HR suppression effects of *ZmPGH1* and *ZmQCR7* in the *N. benthamiana* transient expression system were substantially weaker than those of the *HCT1806, HCT4918*, and *CCoAOMT2* genes (Wang and Balint‐Kurti, 2016; He et al., 2019a, 2019b). By the same token, we might expect that it would be difficult to observe phenotypic effects of suppressing the expression of *ZmPGH1* and *ZmQCR7* in maize.

### 2.5 VIGS constructs fail to silence *Pk1b*, *LOX9*, and *SL11* targets in vivo

Not all of the candidate genes chosen for this work could be silenced using the constructs we produced.

*GRMZM2G144042* encodes a protein kinase 1b (Pk1b) and was identified as a candidate gene based on its proximity (816 bp) to a GWAS hit on chromosome 5 at 2,282,955 bp associated with *Rp1‐D21*‐induced HR (Olukolu et al., 2014). Pk1b belongs to the receptor serine/threonine kinase (STK) family and is involved in a signalling cascade. STKs are thought to relay signals derived from LRR‐containing receptors to activate downstream defence responses (Sessa and Martin, 2000; Afzal et al., 2008).

*GRMZM2G017616* encodes a lipoxygenase 9 (LOX9). Included in its sequence is a SNP (chromosome 1, 16,578,218 bp) that was highly associated with variation in *Rp1‐D21*‐induced HR in our previous GWAS (Olukolu et al., 2014). LOX9 belongs to the 13‐lipoxygenases family and was found to have a role in defence against southern leaf blight (*Bipolaris maydis*) in maize (Christensen et al., 2015). It also has a role in jasmonic acid biosynthetic pathways (Porta and Rocha‐Sosa, 2002; Woldemariam et al., 2018). Considering these potential roles in the defence response, we inferred that *GRMZM2G017616* might have a role in regulating *Rp1‐D21*‐induced HR.

*GRMZM2G351387* encodes a homolog of spotted leaf 11 (SL11)/ plant Ubox 13 (PUB13) protein. It is located on chromosome 3, 11.5 kb from a SNP at 126,183,689 bp that was associated with variation in *Rp1‐D21*‐induced HR (Olukolu et al., 2014). Rice SL11 displays E3 ubiquitin ligase activity and was found to negatively regulate plant defence and cell death (Zeng et al., 2004). The *Arabidopsis* ortholog of SL11, called PUB13, was also involved in negative regulation of plant innate immunity by direct ubiquitination of flagellin receptor FLS2 (Lu et al., 2011) and chitin receptor LYK5 (Liao et al., 2017). T‐DNA insertions in *Arabidopsis PUB13* resulted in enhanced cell death, salicylic acid accumulation, and resistance to biotrophic pathogens (Li et al., 2012). It was therefore targeted as a promising candidate gene for regulation of *Rp1‐D21* activity.

The levels of the transcripts of each of these genes, *Pk1b, LOX9*, and *SL11*, were unaffected in B73:*Rp1‐D21* plants infected with the VIGS constructs designed to suppress their expression (Figure S3b). As might be expected, there were also no differences in the severity of the HR caused by *Rp1‐D21* in any of the B73:*Rp1‐D21* infected by these constructs (Figure S3a,c). To further test the role of *Pk1b* in regulating *Rp1‐D21*‐induced HR, we also employed transient expression in *N. benthamiana*. Coexpression of *Pk1b* with *Rp1‐D21* did not significantly affect *Rp1‐D21*‐induced cell death (Figure S4).

Because these genes were not silenced, we are unable to draw any conclusions about their roles in mediating *Rp1‐D21*‐induced cell death. There are several plausible reasons for ineffective silencing. The most common is the instability of the silencing vector leading to the loss of the target gene‐specific insert (Scofield and Nelson, 2009). As described in the materials and methods, we checked each infected plant for retention of the sequence inserted in pFoMV‐V and tried to only select plants for further analysis where we could determine that the insert was retained. The only construct that consistently lost its insert was pFoMV‐*Pk1b*, so for this construct at least we have a simple explanation for the failure to suppress expression. In the other two cases, constructs designed to target other portions of the gene might be more effective. It is hard to predict which regions of the gene to target to achieve the most efficient suppression of expression (Yeom et al., 2012). When selecting the fragment for silencing, our overriding consideration was specificity and size (about 500 bp), as discussed by Liu and Page (2008). Software such as siRNA‐Finder may offer some more sophisticated methods of identifying target sequences for RNAi applications (Lück et al., 2019). Using a VIGS system based on the bean pod mottle virus, Zhang et al. (2010a) noted that targeting the 3′ end of open reading frames seemed to be more efficient than targeting the 5′ ends. In our case *SL11* and *LOX9* were targeted at the 3′ end but were not silenced (Figure S3). Liu et al. (2016) used inverted repeats to achieve efficient gene silencing in monocots with an independent FoMV‐based VIGS system.

## 3 CONCLUSIONS

Here we have attempted to use the FoMV VIGS system to characterize the roles of 12 genes in modulating the HR induced by the *Rp1‐D21* autoactive R‐protein in maize. In six cases we have observed an effect and we can therefore assign a role to the encoded protein. In six other cases we did not observe an effect and in these cases the conclusions are less obvious. Even in the cases where the expression of the targeted genes was suppressed, we still cannot definitively conclude that the encoded proteins play no role in regulating HR because neither the relationship between RNA and protein levels nor the relationship between protein levels and expected phenotype are clear. This highlights a weakness of the VIGS approach: although the name of the technique implies otherwise, VIGS does not ever completely silence gene expression. Therefore, the role of a gene cannot be definitively ruled out using VIGS even if no phenotypic change is observed. In this study, of our 12 constructs, three provided no detectable suppression of mRNA levels of the targeted genes while the other nine gave various levels of suppression ranging from 1.63‐ to 16.83‐fold. The reasons for this variability are unclear but are probably due to the secondary structure of the mRNAs and the stability of the specific inserts in the FoMV vector. Other disadvantages of VIGS include the fact that it may be hard to differentiate the effects of genes with very similar sequences and that it is transient and can only be used in immature maize plants.

Nevertheless, we were able to use VIGS to efficiently assess the roles of several candidate genes associated with modulation of HR. In four cases we examined the roles of genes that we had previously validated in a *N. benthamiana* ectopic transient expression system. In two of these cases, the *HCT* and *CCoAOMT* genes, the phenotypes we observed in maize conformed to our expectations. In two others, the *PGH1* and the *QCR7* genes, we did not observe the expected effect. While, as discussed above, we cannot draw definitive conclusions in these later cases, it is likely that the lack of effect observed for *PGH1* and *QCR7* may be due to a combination of lower gene suppression (2.46‐ and 2.08‐fold, respectively) compared to *HCT* and *CCoAOMT* (10.52‐ and 4.16‐fold) and the fact that their effect on HR is more subtle in any case, as demonstrated in the *N. benthamiana* system (He et al., 2019a, 2019b). In addition, we were able to implicate two previously uncharacterized genes, *VPS37* and *IQM3*, in the modulation of *Rp1‐D21*‐induced HR.

We also examined the role of the HSP90/SGT1/RAR1 chaperone complex. Scofield et al. (2005) used the barley stripe mosaic virus (BSMV) VIGS system to show that silencing homologs of each of these three genes in wheat compromised resistance to leaf rust conferred by the *Lr21* NLR resistance gene. As discussed above, several other studies have also demonstrated similar effects of these genes on NLR‐mediated resistance and HR in other studies. In our experiments we found that suppressing expression of *HSP90* homologs suppressed *Rp1‐D21*‐induced HR as expected but suppressing *RAR1* expression had no effect on HR while suppressing *SGT1* expression actually enhanced the HR phenotype. Further work will be required to deconvolute these effects. Interpretation is complicated by the fact that each of these genes are members of small gene families in maize. In each case our constructs were designed to suppress expression of all functional homologs of each gene, five *HSP90* homologs, two *SGT1* homologs, and two *RAR1* homologs. With *SGT1* we were able to use the *N. benthamiana* system to show that the one of the two *SGT1* homologs, *SGT5019*, was probably important for modulating *Rp1‐D21* activity.

In conclusion, this study demonstrates the feasibility of performing high‐throughput functional genomics in maize using FoMV VIGS. Using this system, we can produce robust evidence to validate GWAS predictions in the endogenous maize system rather than using a proxy heterologous *N. benthamiana* expression system as we had previously. Additionally, this approach could be easily adapted for the characterization of the numerous other lesion phenotypes identified in maize (Johal, 2007).

## 4 MATERIALS AND METHODS

### 4.1 Plant materials and virus infection procedures

The B73:*Rp1‐D21* line, the commonly used B73 line into which the *Rp1‐D21* gene has been introgressed through multiple backcrosses, was used for all experiments. B73:*Rp1‐D21* is maintained by crossing a B73:*Rp1‐D21* plant heterozygous for *Rp1‐D21* to B73, and therefore it segregates at a 1:1 ratio for the presence/absence of the *Rp1‐D21* mutant allele. When “B73:*Rp1‐D21* plants” are referred to in this study, it always refers to the segregants that are heterozygous for *Rp1‐D21* and display the spontaneous lesion phenotype.

All VIGS experiments used a standardized growth and screening timeline (Figure S5). All VIGS experiments used at least three individual plants for each treatment and were repeated three times. Seeds were treated with 1% vol/vol H_2_O_2_ for 2 min and incubated in water containing 2 mg/ml calcium sulphate to enhance germination. Seeds were germinated using water‐imbibed germination paper for 6 days in a growth chamber set to 22/18°C using a 16:8 hr photoperiod. Methods for biolistic inoculation of FoMV DNA constructs were derived from a prior publication with amendment (Mei and Whitham, 2018). Among 6‐day‐old seedlings grown in germination paper, those possessing an emerged third leaf (V1 stage) or any symptoms of damping off were discarded. Only healthy seedlings possessing an extended cotyledon and first true leaf were used for biolistics. Biolistics were carried out using a Biolistic PDS‐1000/HE Particle Delivery System located in the North Carolina State University (NCSU) Phytotron. Bombarded seedlings were sown in 6‐inch pots containing standard mix (33% Sunshine Redi‐Earth Pro Growing Mix [Canadian Sphagnum] peat moss 50%–65%, vermiculite, dolomitic lime, 0.0001% silicon dioxide, and 66% pea gravel) and grown for an additional 2 weeks using a 12:12 hr 26/22°C day/night cycle with a 2 hr night interruption for c.2 weeks at the NCSU phytotron prior to sample collection. Twenty days after seed germination, the fourth leaves of plants were removed and used to collect SPAD measurements, sampled for RNA isolation, and electronically scanned to collect information about visual phenotypes.

The plants used for data collection and downstream analysis underwent a selection process to distinguish genetic background and successful viral infection. Bombarded B73:*Rp1‐D21* plants used for this work were screened for the presence of *Rp1‐D21* based on the appearance of HR lesions on cotyledons 10 days after germination. Bombarded plants were additionally screened based on the visible presence of FoMV infection (a slightly discolored mosaic pattern that was most notable at the leaf tip) and marked between 13 and 15 days after germination. This was done to distinguish plants that were infected from those that failed to infect after bombardment. This had to be done at 13–15 days because later in development *Rp1‐D21*‐associated lesions tended to mask the visible symptoms of FoMV infection.

*N. benthamiana* plants were grown under artificial lighting in a growth chamber at 23°C for a 16 hr light:8 hr dark photoperiod. Transient assays were performed using 4‐week‐old *N. benthamiana* plants.

### 4.2 Construction of FoMV VIGS vectors

VIGS inserts were amplified using B73 cDNA as a template. Total RNA was isolated from maize leaf using TRIzol reagent (Invitrogen) according to the manufacturer's protocol. cDNA was synthesized from a total RNA template (3 µg) using RevertAid reverse transcriptase (Thermo Scientific). This cDNA was used for amplification of the maize candidate genes shown in Table 1. PCR was performed using each primer pair listed in Table S1, and the product was digested with *Xba*I and *Xho*I for insertion in the antisense orientation into pFoMV‐V to generate the candidate gene‐silencing constructs. Digested products were purified using a silica column (DNA Clean & Concentrator‐5; Zymo Research) and eluted in 6 µl of elution buffer. Purified inserts were ligated with pFoMV‐V digested by the same enzymes using T4 DNA ligase. Ligated product was transformed to *Escherichia coli* DH5α by a heat shock method. Resulting clones were sequenced by the Genomic Sciences Laboratory laboratory at NCSU.

### 4.3 Measurement of SPAD

SPAD measurements were taken using a Minolta chlorophyll meter (Agriculture Solutions LLC). The leaf‐clip sensor was attached to the fourth leaf, and three readings per leaf were measured. A minimum of 18 readings across six leaves were recorded for each treatment. The total chlorophyll content (µg/cm^2^) was calculated using the following formula (Cerovic et al., 2012):

$$\text{Total chlorophyll content}\;=\;\left(99\times \;\text{mean SPAD data}\right)/\left(144‐\;\text{mean SPAD data}\right)$$

The statistical significance between treatments was determined using one‐way analysis of variance (ANOVA) at *p* < .05.

### 4.4 RNA isolation and RT‐qPCR

RNA from infected maize was extracted from the lower half of the fourth leaf used for SPAD measurements. One hundred milligrams of leaf tissue samples was collected using a 3 mm biopsy punch. RNA was isolated using TRIzol (Invitrogen) and cDNA was synthesized using a Revert Aid First Strand cDNA synthesis Kit (Thermo Scientific). RNA material from plants presenting a visible FoMV infection were then screened for the presence of VIGS inserts using primer pair FM‐5840F and FM‐6138R (Table S2). With the exception of pFoMV‐*Pk1b* infections, only samples displaying the correctly amplified insert were further analysed by RT‐qPCR. Real‐time qPCR was conducted using iTaq Universal SYBR Green Supermix (Bio‐Rad) and StepOne Plus (Applied Biosystems). The primers used are provided in Table S1. Fold change was calculated using 2^−ΔΔ^ *^C^* ^t^ (Livak and Schmittgen, 2001). The statistical significance between treatments was determined using one‐way ANOVA at *p* < .05. RNA sampling and RT‐qPCR were performed for at least six individual plants per control or experimental group. Two technical replicates were used for each of three biological replicates.

### 4.5 Construction of clones for transient studies in *N. benthamiana*

The purified PCR products of *SGT5019*, *SGT9704*, and *Pk1b* amplified from maize B73 cDNA were cloned into pCR8/GW/TOPO TA vector following the manufacturer’s protocol (Thermo Fisher Scientific). The resulting ligation mixtures were transformed into *E. coli* and positive clones were identified by colony PCR. Positive clones were then subcloned into pGWB641 featuring a recombinant C terminal EYFP tag (Nakamura et al., 2010) using LR clonase II (Thermo Fisher Scientific). Positive clones were then transformed into *Agrobacterium tumefaciens* GV3101.

### 4.6 Transient expression in *N. benthamiana*

*A. tumefaciens* GV3101 (pMP90) carrying recombinant clones was grown at 28°C overnight in 50 ml of lysogeny broth (LB) with appropriate antibiotics. Cultures were centrifuged at 3,400 ×g for 30 min at 4°C to obtain a pellet. The resultant pellet was resuspended in infiltration buffer and diluted to an optical density of 600 nm (OD_600_) = 0.6. GV3101 (pMP90) carrying a helper plasmid expressing TBSV p19 (helper plasmid) at OD_600_ = 0.3 was added to each pellet suspension. The resultant mixture was used for transient expression in *N. benthamiana* as previously performed (Wang et al., 2015a).

### 4.7 Ion leakage assays

Five leaf discs (1.5 cm diameter) were collected from at least three leaves on three separate *N. benthamiana* plants for each treatment and incubated in 5 ml of sterilized distilled water at 100 rpm for 3 hr at room temperature. The initial conductivity (C1) was measured using a conductivity meter (model 4403; Markson Science, Inc.). Samples were boiled at 95°C for 15 min and final conductivity was measured (C2). Ion leakage was obtained by calculating the C1:C2 ratio. Statistical analysis was performed using one‐way ANOVA at *p* < .05.

### 4.8 Western blotting

At least three leaf discs (1.2 mm diameter) were collected from the area infiltrated with *A. tumefaciens* in each *N. benthamiana* leaf at 30 hr postinfiltration (hpi) for protein expression analysis. Total protein was isolated using 300 µl of protein extraction buffer (20 mM Tris‐HCl, pH 8.0, 150 mM NaCl, 1 mM EDTA, pH 8.0, 1% Triton X‐100, 0.1% sodium dodecyl sulphate (SDS), 10 mM dithiothreitol, 40 mM MG132, and 1 × plant protein protease inhibitor mixture; Sigma‐Aldrich). Protein sample preparation and SDS polyacrylamide electrophoresis was carried out as previously described (Wang et al., 2015b). Protein gels were transferred to nitrocellulose and incubated with anti‐HA antibody conjugated with horseradish peroxidase (HP; Roche) at 1:350 dilution. GFP was detected using a 1:6,000 dilution of anti‐GFP (monoclonal, mouse; Abcam) followed by incubation with 1:15,000 of goat anti‐mouse antibody conjugated with horseradish peroxidase (HRP) (Sigma). The HRP signal was developed using SuperSignal West Femto Maximum Sensitivity Substrate (Thermo Scientific) and detected by chemiluminescence.

## Supporting information


**FIGURE S1** Images of B73 plants infected with the pFoMV constructs indicated. The B73 plants were wild‐type segregants from the B73:Rp1‐D21 line which segregates 1:1 for the presence of *Rp1‐D21*. Typical symptoms of FoMV infection are observed; FoMV infection of B73 results in diffuse and subtle discoloration of the leaf blade in a mosaic pattern that is concentrated most at the leaf tip. Characteristic bleaching stripes associated with *PDS* suppression by VIGS are observed in leaves infected with pFoMV‐*PDS*. A bleached streaking pattern is also evident in leaves infected with pFoMV‐*HSP90*

**FIGURE S2** Infection of B73:Rp1‐D21 by pFoMV‐V and pFoMV‐*PDS*. (a) Image of fourth leaves from inoculated plants 14 dpi. Black squares outline the area that is shown magnified directly below the main picture. (b) Real‐time RT‐qPCR analysis of *PDS* in B73:Rp1‐D21. Significant suppression of *PDS* transcripts is detected in the fourth leaves of plants that were infected with pFoMV*‐PDS*. Value was normalized using *actin* as a reference. (c) SPAD data from inoculated plants. Significant decrease in chlorophyll content is observed in systemic leaves of plants that infected with pFoMV‐PDS compared with pFoMV‐V. (*p < .05, ***p < .001). The value is derived from at least three biological replications

**FIGURE S3** Infection of B73:Rp1‐D21 using pFoMV‐*Pk1b*, pFoMV‐*SL11*, and pFoMV‐*LOX9*. (a) Image of fourth leaves from inoculated plants 14 dpi. Black squares outline the area that is shown magnified directly below the main picture. (b) Real‐time RT‐qPCR analysis of *Pk1b*, *SL11*, and *LOX9* in B73:Rp1‐D21. Values were normalized using *actin* as a reference. (c) SPAD data from inoculated plants. (*p < .05 compared with the empty vector by ANOVA; ns, not significant). Values are derived from at least three biological replications

**FIGURE S4** Transient expression of protein kinase 1b (Pk1b) in *Nicotiana benthamiana*. (a) Transient coexpression of *Pk1b* with *Rp1‐D21* in *N. benthamiana*. GUS was used as a negative control. *Agrobacterium* carrying each construct was diluted to a final concentration of OD_600_ =1.0. Leaves were harvested 3 dpi. Regions of infiltration were marked with ovals. (b) Ion leakage conductivity was measured at 36 hr after co‐expression of Pk1b or GUS with *Rp1‐D21*. HCT1806 was used as a positive control for suppression of *Rp1‐D21*‐induced HR. Data represent means ± *SD* from three independent plants. (**p < .01 compared with *Rp1‐D21* by ANOVA; ns, not significant)

**FIGURE S5** Experimental timeline. All infection experiments were performed according to a 20‐day timeline. B73:*Rp1‐D21* seeds were imbibed in water and germinated in paper. The resulting seedlings were used for biolistic introduction of virus constructs at 6 days and then immediately potted in soil. At 4 dpi (10 days) the appearance of *Rp1‐D21* lesions was noted and at 7–9 dpi (13–15 days), evidence of viral infection was visible and used to select plants for phenotyping and data collection. Imaging and sample collection for molecular analysis was performed at 14 dpi (20 days)

**TABLE S1** Primers used to amplify fragments for silencing constructs

**TABLE S2** Primers used for RT‐qPCR

## Data Availability

The data that support the findings of this study are available from the corresponding author upon reasonable request.
