# Supplementary figures and images for: Use of virus‐induced gene silencing to characterize genes involved in modulating hypersensitive cell death in maize
Source: Mol Plant Pathol. 2020 Oct 10;21(12):1662–76. doi: 10.1111/mpp.12999 (PMC7694674; doi:10.1111/mpp.12999)

Figure S1

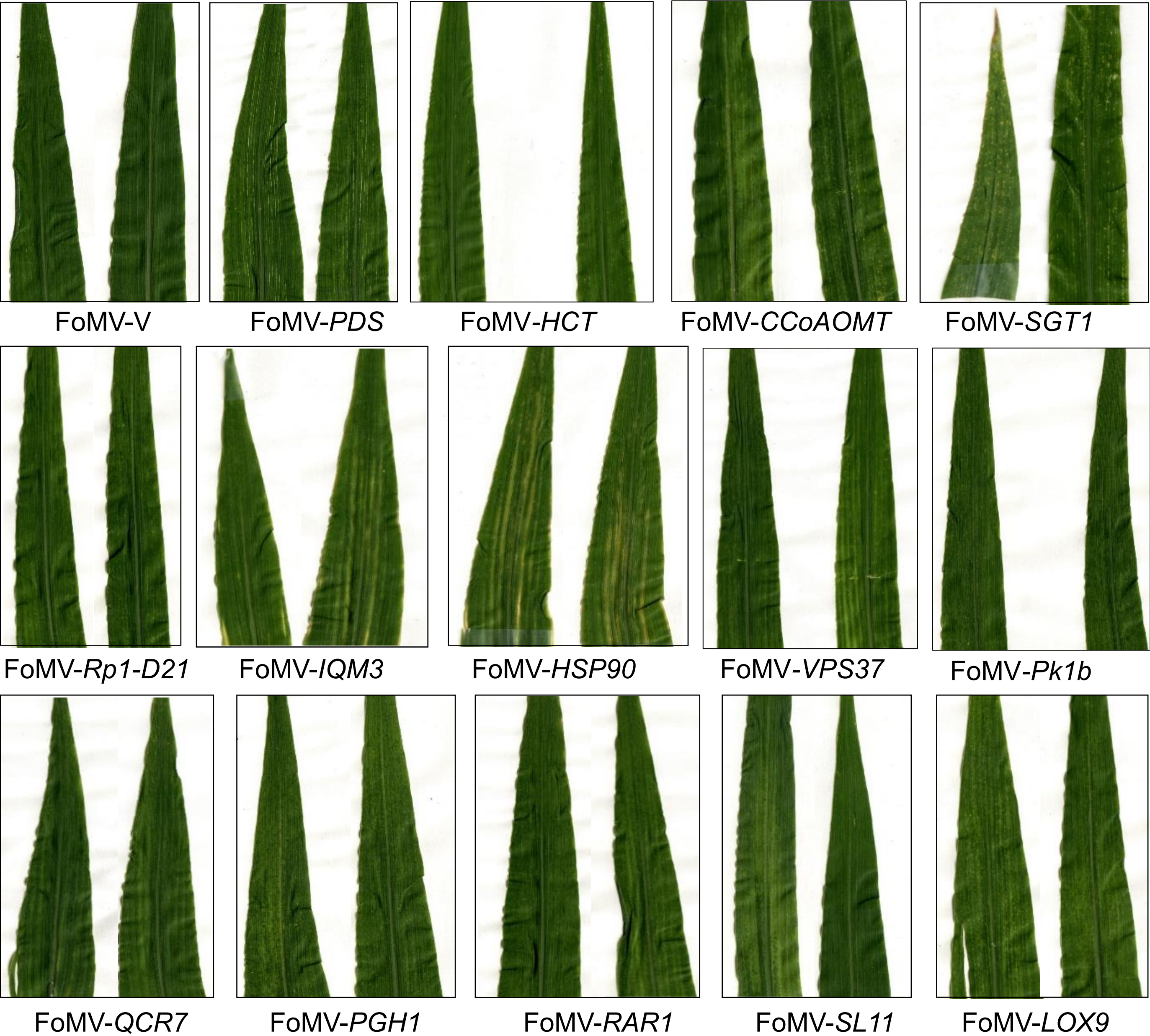

Supplement: Supplementary file 1 — FIGURE S1 Images of B73 plants infected with the pFoMV constructs indicated. The B73 plants were wild‐type segregants from the B73:Rp1‐D21 line which segregates 1:1 for the presence of Rp1‐D21. Typical symptoms of FoMV infection are observed; FoMV infection of B73 results in diffuse and subtle discoloration of the leaf blade in a mosaic pattern that is concentrated most at the leaf tip. Characteristic bleaching stripes associated with PDS suppression by VIGS are observed in leaves infected with pFoMV‐PDS. A bleached streaking pattern is also evident in leaves infected with pFoMV‐HSP90 [file MPP-21-1662-s001.pdf]

Figure S2

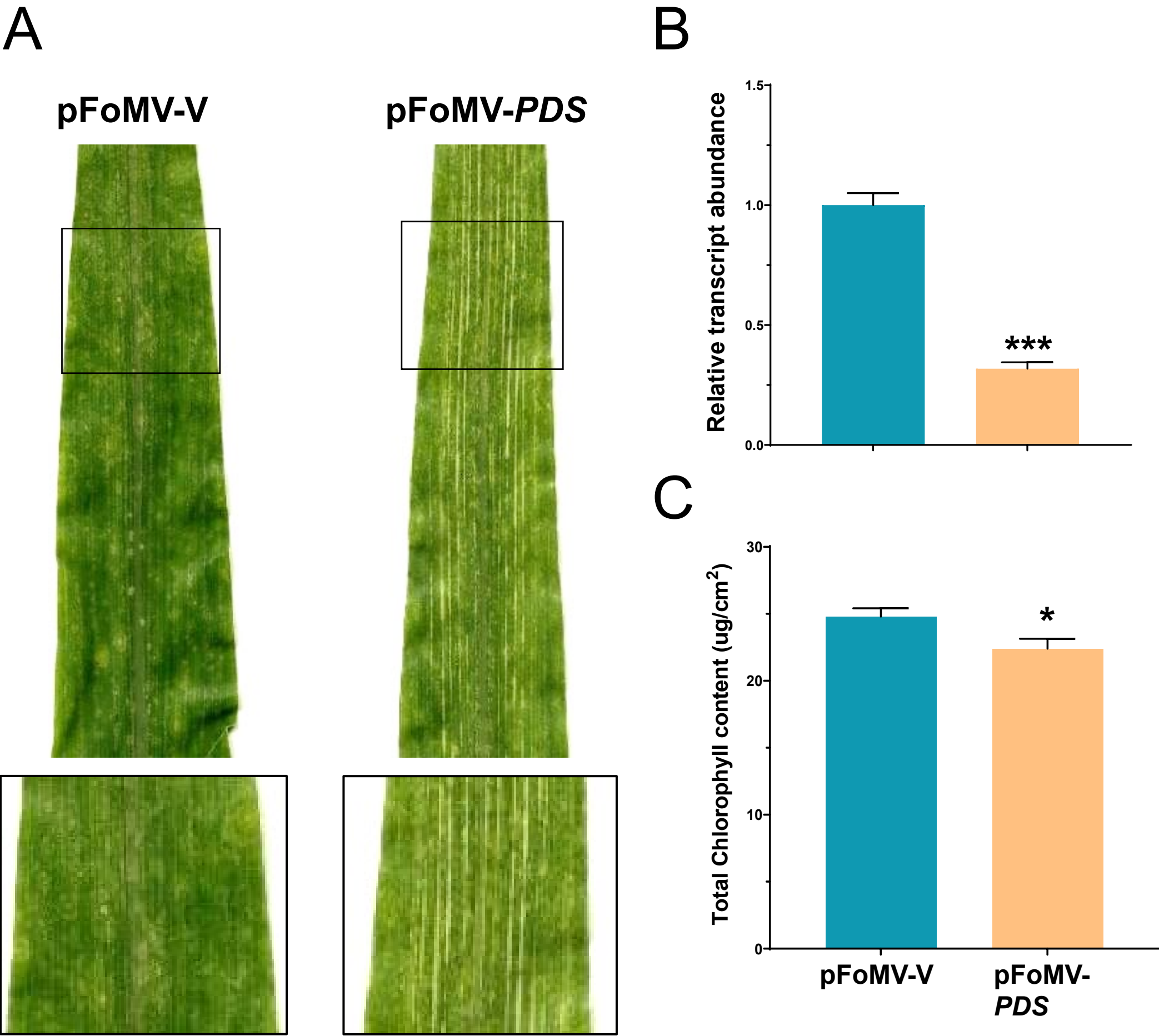

Supplement: Supplementary file 2 — FIGURE S2 Infection of B73:Rp1‐D21 by pFoMV‐V and pFoMV‐PDS. (a) Image of fourth leaves from inoculated plants 14 dpi. Black squares outline the area that is shown magnified directly below the main picture. (b) Real‐time RT‐qPCR analysis of PDS in B73:Rp1‐D21. Significant suppression of PDS transcripts is detected in the fourth leaves of plants that were infected with pFoMV‐PDS. Value was normalized using actin as a reference. (c) SPAD data from inoculated plants. Significant decrease in chlorophyll content is observed in systemic leaves of plants that infected with pFoMV‐PDS compared with pFoMV‐V. (*p < .05, ***p < .001). The value is derived from at least three biological replications [file MPP-21-1662-s002.pdf]

Figure S3

A

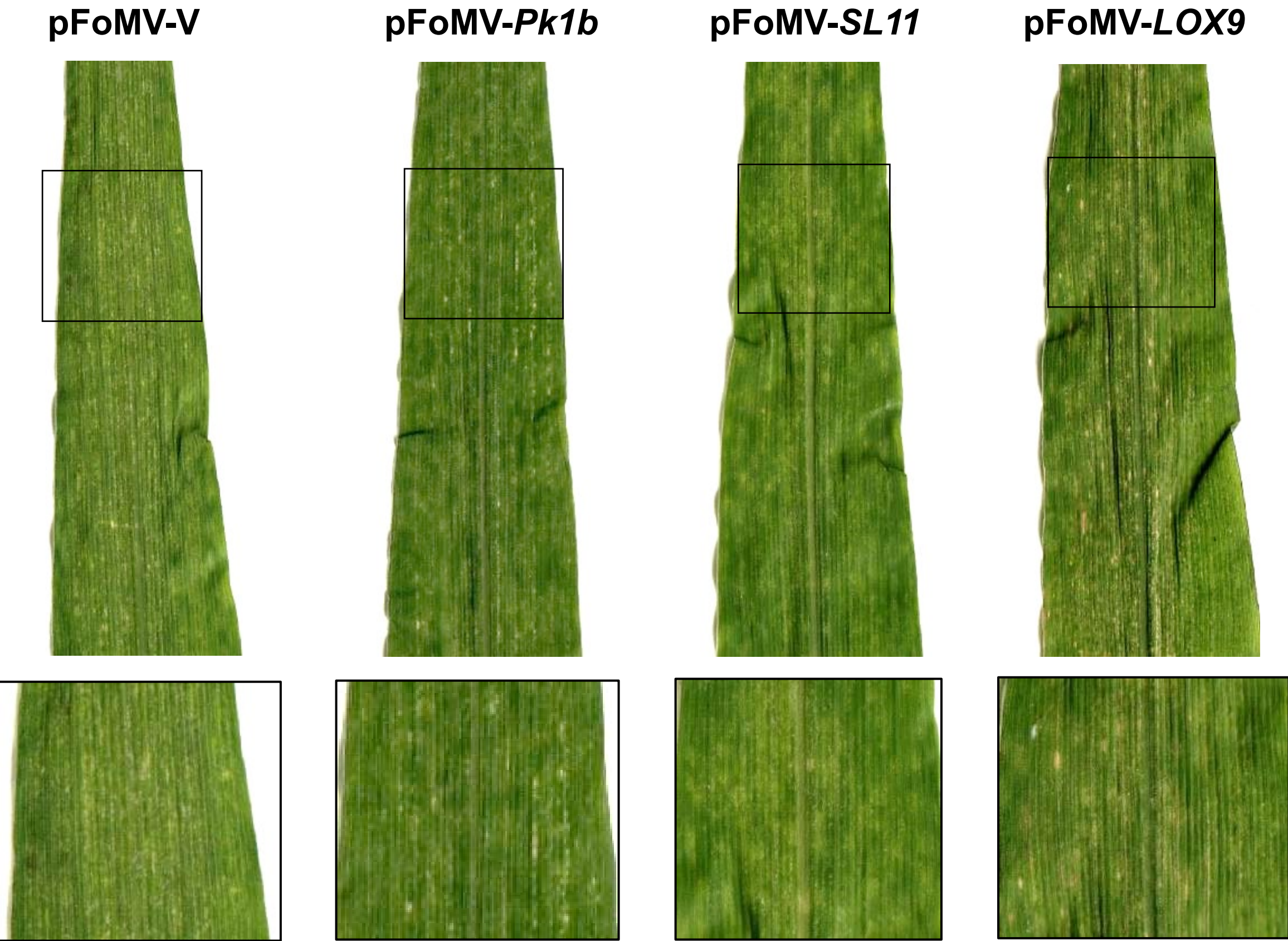

B

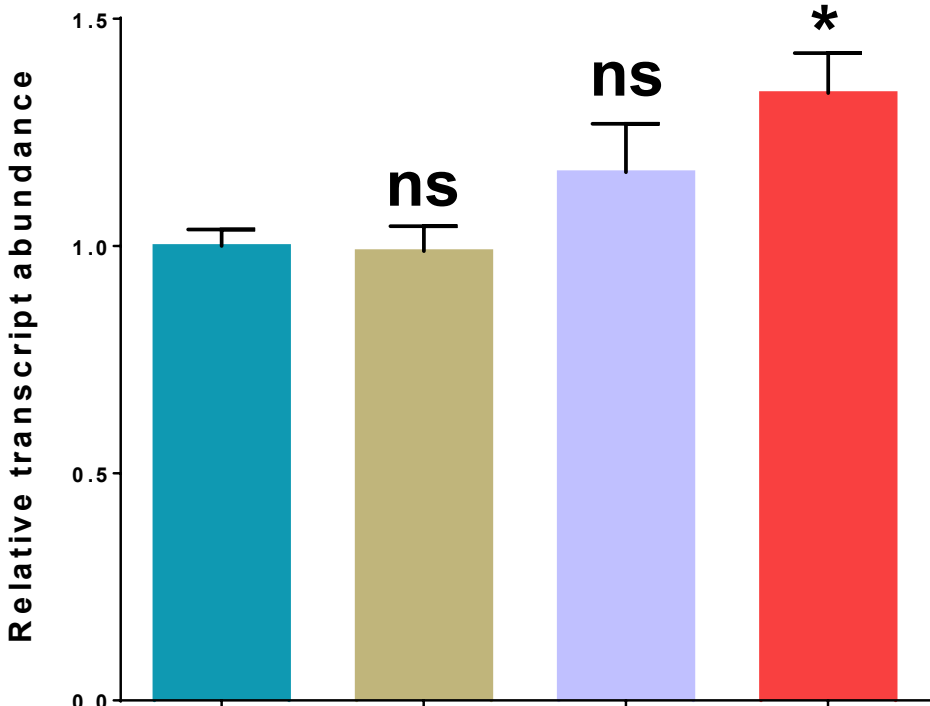

C

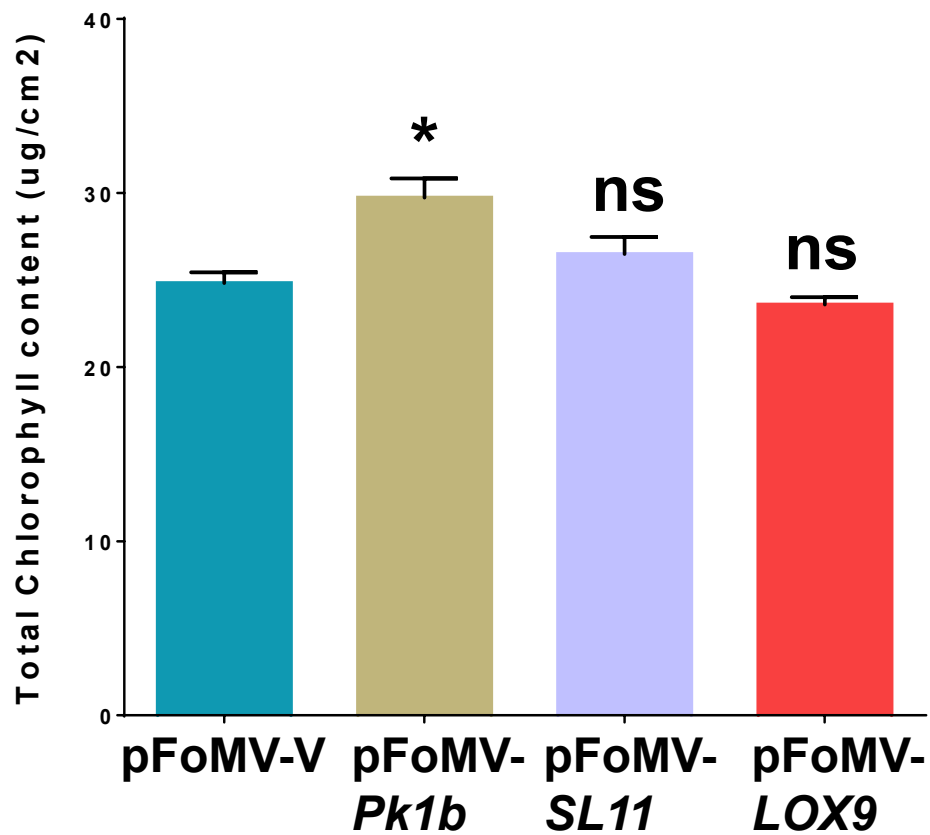

Supplement: Supplementary file 3 — FIGURE S3 Infection of B73:Rp1‐D21 using pFoMV‐Pk1b, pFoMV‐SL11, and pFoMV‐LOX9. (a) Image of fourth leaves from inoculated plants 14 dpi. Black squares outline the area that is shown magnified directly below the main picture. (b) Real‐time RT‐qPCR analysis of Pk1b, SL11, and LOX9 in B73:Rp1‐D21. Values were normalized using actin as a reference. (c) SPAD data from inoculated plants. (*p < .05 compared with the empty vector by ANOVA; ns, not significant). Values are derived from at least three biological replications [file MPP-21-1662-s003.pdf]

Figure S4

A

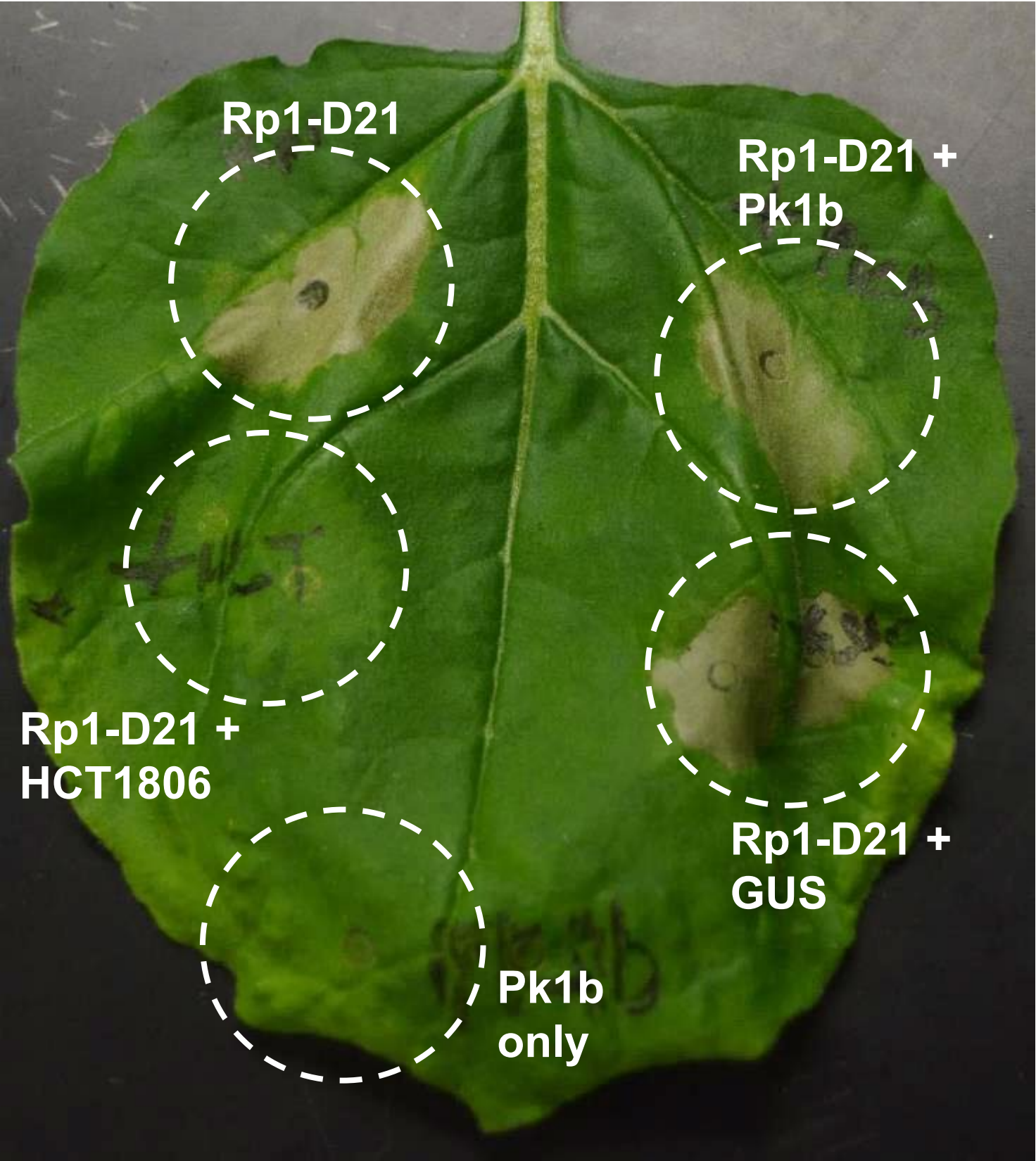

B

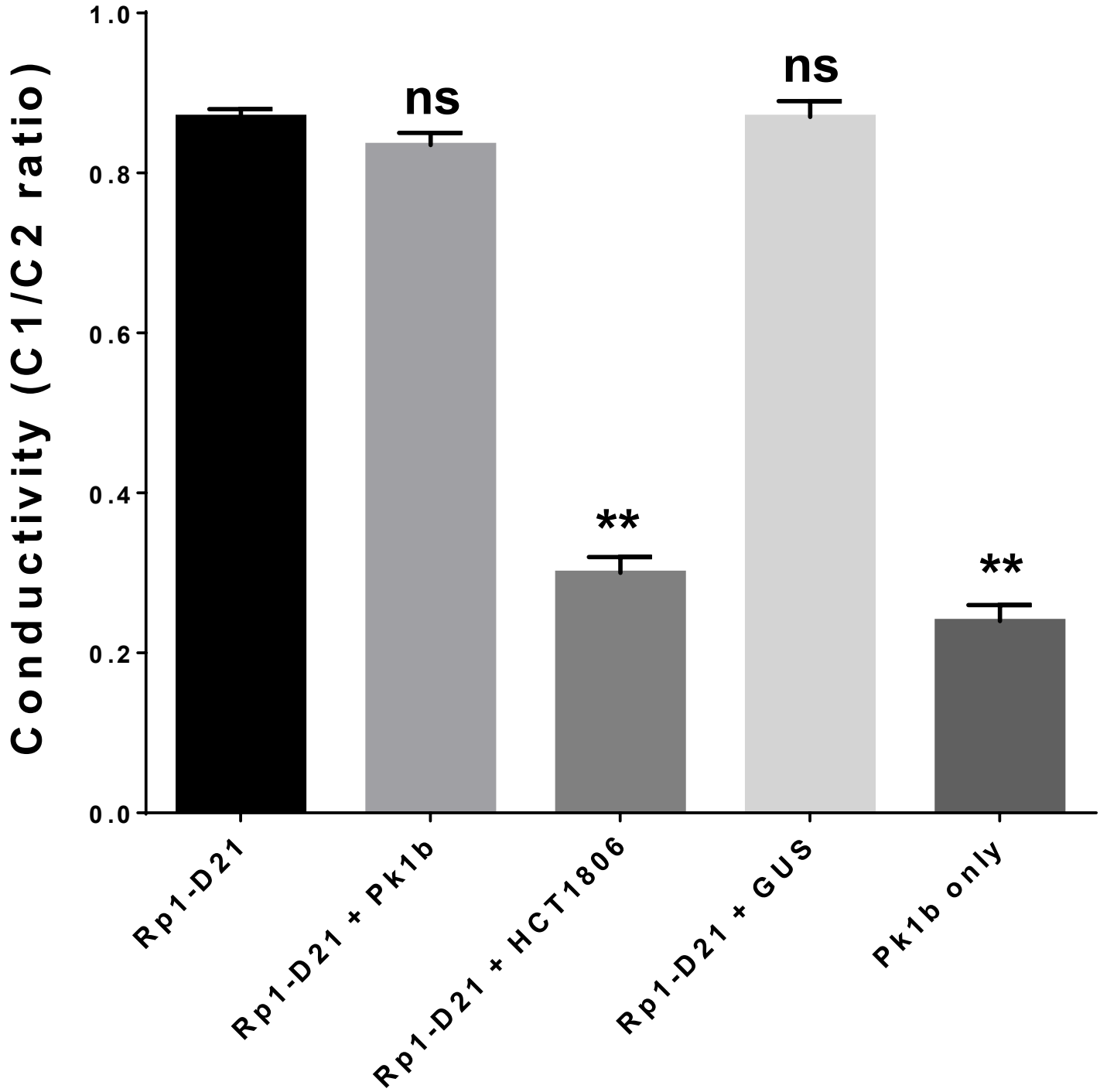

Supplement: Supplementary file 4 — FIGURE S4 Transient expression of protein kinase 1b (Pk1b) in Nicotiana benthamiana. (a) Transient coexpression of Pk1b with Rp1‐D21 in N. benthamiana. GUS was used as a negative control. Agrobacterium carrying each construct was diluted to a final concentration of OD600 =1.0. Leaves were harvested 3 dpi. Regions of infiltration were marked with ovals. (b) Ion leakage conductivity was measured at 36 hr after co‐expression of Pk1b or GUS with Rp1‐D21. HCT1806 was used as a positive control for suppression of Rp1‐D21‐induced HR. Data represent means ± SD from three independent plants. (**p < .01 compared with Rp1‐D21 by ANOVA; ns, not significant) [file MPP-21-1662-s004.pdf]

Figure S5

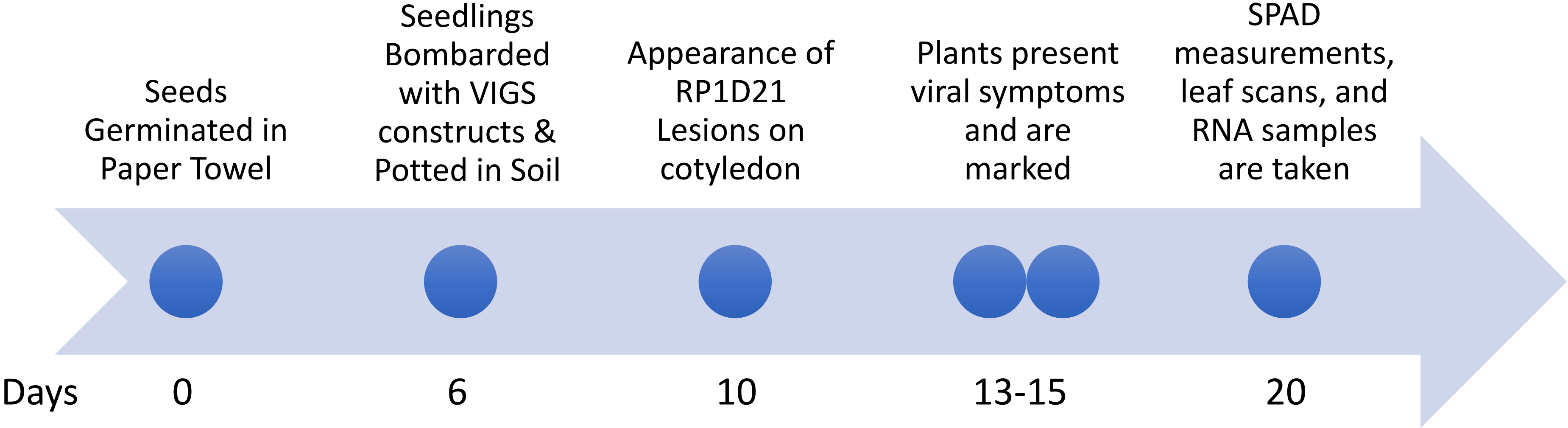

Supplement: Supplementary file 5 — FIGURE S5 Experimental timeline. All infection experiments were performed according to a 20‐day timeline. B73:Rp1‐D21 seeds were imbibed in water and germinated in paper. The resulting seedlings were used for biolistic introduction of virus constructs at 6 days and then immediately potted in soil. At 4 dpi (10 days) the appearance of Rp1‐D21 lesions was noted and at 7–9 dpi (13–15 days), evidence of viral infection was visible and used to select plants for phenotyping and data collection. Imaging and sample collection for molecular analysis was performed at 14 dpi (20 days) [file MPP-21-1662-s005.pdf]
