# Supplementary material for: Use of virus‐induced gene silencing to characterize genes involved in modulating hypersensitive cell death in maize
Source: Mol Plant Pathol. 2020 Oct 10;21(12):1662–76. doi: 10.1111/mpp.12999 (PMC7694674; doi:10.1111/mpp.12999)
Supplement: Supplementary file 6 — TABLE S1 Primers used to amplify fragments for silencing constructs [file MPP-21-1662-s006.docx]

Supplementary table S1. Primers used to amplify fragments for silencing constructs

| **Gene ID (B73 v3.)** | **Forward** | **Reverse** | **Size of amplified product (bp)** |
| --- | --- | --- | --- |
| Rp1-D21 | CTCGAGCTAGGAGCGTACTCATCGTAC | TCTAGAGCTCTGTATCTGGTGGTAGG | 469 |
| GRMZM2G012631 | CTCGAG GAAGCACTCCGAGTTCATCAG | TCTAGA CTCTTCGCAGTTGTCCATGATG | 454 |
| GRMZM2G017616 | CTCGAGAGTCGTATGTGCAGCTCTACTAC | TCTAGACACTCATTCATATGGAGATGCTG | 654 |
| GRMZM2G023575 | CTCGAGCCTAATTGCAATCCGCGTAAAC | TCTAGAGTGTAGACTAGCACTAGAAGAG | 307 |
| GRMZM2G061806 | CTCGAGTAGAATGGAGACCATGAAGGC | TCTAGAGAAGAAGGCGCTCATGGAGT | 502 |
| GRMZM2G099363 | CTCGAGAGCTCATCGGCGCCAAGAAGAC | TCTAGAGTAGAAGCGGATGTACTTGCGCATG | 410 |
| GRMZM2G105019 | CTCGAGTGGTGAACAGATGTTGAGTGT | TCTAGACCACTTCTTGAGCTCCATACC | 505 |
| GRMZM2G135763 | CTCGAGAGAGAATGTTCATGTCTGGGA | TCTAGACATTGTCAGAAACTGTAGTCG | 477 |
| GRMZM2G144042 | CTCGAGATGATTTTCCAAGCTCCCGC | TCTAGAGGAGGGTTCGGCTCTGTCTA | 258 |
| GRMZM2G318346 | CTCGAGCACGCAGTGGTGCATTGAC | TCTAGACGTGCATGTTCCACGCTC | 360 |
| GRMZM2G351387 | CTCGAGGCGATCCTGTCGATACTGTC | TCTAGAGACATCAGCCTCCGTAACG | 387 |
| GRMZM2G439311^1^ | CTCGAGAAGAAGGAGACGGTGCTGTG | TCTAGAGATAAGCTGGCGCCTCCC | 122 |
| GRMZM5G868908 | CTCGAGGCATGAAAGAATGGAGCTGC | TCTAGAATCACAGCATTTCCACCCTCTC | 455 |

^1^Gene annotation is not correct in B73 genome v4. We designed the primers based on our sequence from amplified PCR product
