# Supplementary material for: Use of virus‐induced gene silencing to characterize genes involved in modulating hypersensitive cell death in maize
Source: Mol Plant Pathol. 2020 Oct 10;21(12):1662–76. doi: 10.1111/mpp.12999 (PMC7694674; doi:10.1111/mpp.12999)
Supplement: Supplementary file 7 — TABLE S2 Primers used for RT‐qPCR [file MPP-21-1662-s007.docx]

Supplementary table S2. Primers used for RT-qPCR

| **Gene ID (B73 v3.)** | **Forward** | **Reverse** |
| --- | --- | --- |
| Rp1-D21 | ATTGGAAGTGTTCTGGTTCC | GCTTCATCTGTAATACTGCATG |
| GRMZM2G012631^1^ | GCATTGGTATGACCAAGTC | TACTGCTCATCATCATTGTGC |
| GRMZM2G017616 | GTGCTGCCGTACGAGCTG | GTGTATTCTCTACAAGTGCTG |
| GRMZM2G023575 | CAGACATCACTCCGCTGAC | CAGGACAGAGTACATAAGACG |
| GRMZM2G061806 | GCACTCCTGCAGATCAAGCTC | GAACGCTGAAGTCCTTGCC |
| GRMZM2G099363 | CGCGCAAGCCAGTGCCGCAC | CTGCGCCGGTGCAGCCTTG |
| GRMZM2G105019^2^ | GGAATCAAATGGCACTGTTCTC | GGTGGACGGAATTAGGTCATA |
| GRMZM2G126010 (actin) | GGTTTCGCTGGTGATGATGC | CAATGCCATGCTCAATCGGG |
| GRMZM2G135763 | CTGCTACATTTCTGTTGGTGATG | TCCCAGACATGAACATTCTCT |
| GRMZM2G144042 | ACGAAGCTAAGATGAGGCCG | CCAACAGTCGTCTCCTTCGT |
| GRMZM2G318346 | CTCTCCATGAAGCATCAGTAC | GCATGCATTCTGATTTGCCG |
| GRMZM2G351387 | GTGGTGGACGAATACAAGGTC | CATGACGAGTGGCACGAGG |
| GRMZM2G439311^3^ | CCTCTGGTGATGGGGTTTGT | GATAAACCCTGGCCCACCT |
| GRMZM5G868908 | GAGAGGGTGGAAATGCTGTGAT | TCACACTTCCAAATGAAATCCTC |
| GRMZM2G410515^4^ (PDS) | CAGCATTGAACGGTTTGGGTCA | TGGAGAAGTTGGTGGGAGTTCC |
| Foxtail mosaic virus^5^ | TCTGTACCGTACGATGAGCCC | GCTGCGTTACTGTTAGGTCG |
|  |  |  |

^1^Primers amplify all 5 HSP90 homologs

^2^Primers amplify both SGT1 homologs

^3^Gene annotation is incorrect in B73 genome v4. We designed the primers based on our sequence from amplified PCR product

^4^Primers are from Mei et al.,2016

^5^Foxtail mosaic virus: FM-5840F and FM-6138R are used for insertion from infected plants (Mei et al., 2016).
